# Effects of high-intensity interval training compared to moderate-intensity continuous training on maximal oxygen consumption and blood pressure in healthy men: A randomized controlled trial

**DOI:** 10.7705/biomedica.4451

**Published:** 2019-09-01

**Authors:** Víctor Hugo Arboleda-Serna, Yuri Feito, Fredy Alonso Patiño-Villada, Astrid Viviana Vargas-Romero, Elkin Fernando Arango-Vélez

**Affiliations:** 1 Grupo de Investigación en Actividad Física para la Salud, Instituto de Educación Física, Universidad de Antioquia, Medellín, Colombia Universidad de Antioquia Grupo de Investigación en Actividad Física para la Salud Instituto de Educación Física Universidad de Antioquia Medellín Colombia; 2 Department of Exercise Science and Sport Management, Kennesaw State University, Kennesaw, GA, USA Kennesaw State University Department of Exercise Science and Sport Management Kennesaw State University KennesawGA USA

**Keywords:** High-intensity interval training, blood pressure, exercise, cardiorespiratory fitness, randomized controlled trial, entrenamiento con intervalos de gran intensidad, presión sanguínea, ejercicio, capacidad cardiovascular, ensayo clínico controlado aleatorio

## Abstract

**Introduction:**

Aerobic exercise generates increased cardiorespiratory fitness, which results in a protective factor for cardiovascular disease. High-intensity interval training (HIIT) might produce higher increases on cardiorespiratory fitness in comparison with moderate-intensity continuous training (MICT); however, current evidence is not conclusive.

**Objective:**

To compare the effects of a low-volume HIIT and a MICT on maximal oxygen consumption (VO2max), systolic blood pressure, and diastolic blood pressure during eight weeks in healthy men between 18 and 44 years of age.

**Materials and methods:**

We conducted a randomized controlled trial. Forty-four volunteers were randomized to HIIT (n=22) or MICT (n=22). Both groups performed 24 sessions on a treadmill. The HIIT group completed 15 bouts of 30 seconds (90-95%, maximal heart rate, HRmax), while the MICT group completed 40 minutes of continuous exercise (65-75% HRmax). The study is registered as a clinical trial via clinicaltrials.gov with identifier number: NCT02288403.

**Results:**

Intra-group analysis showed an increase in VO2max of 3.5 ml/kg/min [95% confidence interval (CI) 2.02 to 4.93; p=0.0001] in HIIT and 1.9 ml/kg/min (95% CI -0.98 to 4.82; p=0.18) in MICT. However, the difference between the two groups was not statistically significant (1.01 ml/kg/min. 95% CI -2.16 to 4.18, p=0.52). MICT generated a greater reduction in systolic blood pressure compared to HIIT (median 8 mm Hg; p<0.001). No statistically significant differences were found between the groups for DBP.

**Conclusions:**

Results indicated no significant change in VO2max with a low-volume HIIT protocol versus MICT after 24 sessions. In contrast, MICT provided a greater reduction in systolic blood pressure compared to HIIT

## Resumen

**Introducción.:**

El ejercicio aeróbico incrementa la capacidad cardiorrespiratoria, considerada como factor de protección frente a enfermedades cardiovasculares. El entrenamiento con intervalos de gran intensidad (*High Intensity Interval Training,* HIIT) podría causar mayores incrementos en la capacidad cardiorrespiratoria comparado con el entrenamiento continuo de intensidad moderada, aunque la información actual no es concluyente.

**Objetivo.:**

Comparar los efectos del entrenamiento con intervalos de gran intensidad de bajo volumen y del entrenamiento continuo de intensidad moderada, en el volumen máximo consumido de oxígeno (VO2max), la presión arterial sistólica y la presión arterial diastólica, durante ocho semanas en hombres sanos entre los 18 y los 44 años de edad.

**Materiales y métodos.:**

Se hizo un ensayo clínico controlado con asignación al azar. Se incluyeron 44 voluntarios, 22 a entrenamiento con intervalos de gran intensidad y 22 a uno continuo de intensidad moderada. Ambos grupos hicieron 24 sesiones en tapiz rodante. El primer grupo completó 15 cargas de 30 segundos (90-95 % de la frecuencia cardiaca máxima y, el segundo hizo 40 minutos continuos (65-75 % de la frecuencia cardiaca máxima). El estudio está registrado en clinicaltrials.gov, código: NCT02288403.

**Resultados.:**

El análisis dentro de cada grupo mostró un aumento en el volumen máximo consumido de oxígeno de 3,5 ml/kg por minuto (intervalo de confianza, IC95% 2,02 a 4,93; p=0,0001) con el entrenamiento con intervalos de gran intensidad, y de 1,9 ml/kg por minuto (IC95% -0,98 a 4,82; p=0,18) con el continuo de intensidad moderada. Sin embargo, las diferencias entre grupos no fueron estadísticamente significativas (1,01 ml/kg por minuto; IC95% -2,16 a 4,18; p=0,52). El entrenamiento continuo de intensidad moderada generó una mayor reducción en la presión arterial sistólica, comparado con el de intervalos de gran intensidad (mediana: 8 mm Hg; p<0,001). Por último, no se hallaron diferencias estadísticamente significativas entre grupos en la presión arterial diastólica.

**Conclusiones.:**

Los resultados no evidenciaron diferencias en el efecto sobre el VO 2máx con un protocolo de entrenamiento con intervalos de gran intensidad de bajo volumen, en comparación con el continuo de intensidad moderada. Por el contrario, con este último, la reducción en la presión arterial sistólica fue mayor que con el de intervalos de gran intensidad.

Maximum oxygen consumption (VO2max) is considered the main indicator to evaluate cardiorespiratory fitness ^1^^,^^2^. VO2max is directly related to cardiovascular health and its increase is associated with a reduced risk of death from cardiovascular disease and for all-cause mortality ^3^^-^^5^. Moderate- intensity continuous training (MICT) has been the most widely used method to increase VO2max in the past. However, in recent years, high-intensity interval training (HIIT) methods have been practiced by individuals with different health conditions and its use is increasing ^6^^,^^7^.

Currently, the gain in VO2max achieved with HIIT versus MICT is under discussion. Some studies have shown that HIIT generated faster and more significant adaptations in VO2max when compared to MICT ^8^^-^^14^ while other investigations have found less pronounced increases in VO2max with HIIT, which indicates that some limitations exist with these types of programs ^15^^-^^17^. Nonetheless, it should be noted that the protocols used in these HIIT studies were comprised of short periods of load and more extensive recovery periods compared to other interventions ^9^^,^^10^^,^^14^^,^^18^^,^^19^.

High blood pressure is a common health condition that is associated with increases in the incidence of all-cause mortality and cardiovascular disease. Cornelissen, *et al.*^20^ reported a decrease in systolic blood pressure and diastolic blood pressure of 3.5 mm Hg (95% CI 2.3-4.6) and 2.5 mm Hg (95% CI 1.7-3.2) with aerobic exercise, respectively. Moreover, studies comparing continuous training and HIIT among non-exercisers hypertensive controls reported significant decreases of 8-mmHg for SBP in all groups ^12^, and mean decreases in systolic blood pressure and diastolic blood pressure with HIIT of 12 and 8 mmHg, respectively, compared with continuous workouts that achieved non-significant reductions of 4.5, and 3.5 mm Hg ^21^. Although exercise is a fundamental aspect in the primary prevention, treatment, and control of hypertension, the optimal frequency, intensity, time, and type of exercise to reduce systolic blood pressure and diastolic blood pressure values are still unclear ^22^.

Therefore, the primary objective of this study was to compare the effect of a low-volume HIIT program versus a MICT program in VO2max among healthy men. The secondary objective was to identify the effect of both exercise programs on systolic and diastolic blood pressure. We hypothesize that those in the HIIT group would have significantly greater improvements in VO2max and BP responses compared to the MICT group.

## Materials and methods

This two-arm randomized control trial with parallel groups was developed following the CONSORT statement for randomized trials of non- pharmacological treatment ^23^ and it is registered as a clinical trial under identifier number NCT02288403.

Participants were recruited via posters, word of mouth, social media, and email around the academic community of a public university in Medellín, Colombia. We asked men between 18 and 44 years of age who did not meet the physical activity recommendations of 150 minutes of aerobic exercise per week to participate. Those who responded and agreed to participate voluntarily in the study were asked to sign an informed consent form. The *Universidad de Antioquia* Research Ethics Committee approved all forms and study protocols.

Individuals with any of the following characteristics were excluded from the study: Those who practiced HIIT, smoked, had a history of pulmonary, metabolic or cardiovascular disease, arrhythmias, heart failure, hypertension, diabetes mellitus, were being treated with anticoagulants, beta-blockers, calcium antagonists, bronchodilators, steroids, or had cognitive, sensory, neuromotor, and/or musculoskeletal disorders that could affect their participation in any of the study protocols. All subjects were evaluated by a sports medicine physician who authorized their participation in the study according to the criteria mentioned above.

For the purpose of this study, we were interested in examining changes in maximal oxygen consumption (VO2max) in the two groups. We evaluated VO2max via a graded exercise test on a treadmill (Trackmaster™, model TMX 425C) using a portable gas analyzer (K4b2, Cosmed Inc., IL, USA).

We were also interested in examining changes in both systolic blood pressure and diastolic blood pressure between the groups. Blood pressures was measured with an Omron M3 HEM-7200-E™ (Omron Healthcare, Co., Ltd., Kyoto, Japan) automatic blood pressure monitor.

A detailed description of the study design along with specific details of the protocols utilized to measure the primary and secondary outcomes have been published elsewhere ^24^. Briefly, participants were randomly assigned to a high-intensity interval training (HIIT) group or a moderate-intensity continuous training (MICT) group. All training sessions for groups were monitored using a heart rate monitor (Polar FT1™; Polar, Lake Success, NY) and supervised by a qualified trainer on alternate days (3 times/week) for eight-weeks. Prior to their assigned exercise session, all participants completed a five-minute warm-up at 50-60% maximal heart rate and completed their respective session with a three-minute cool-down at 40-50% maximal heart rate.

The MICT group was prescribed a 40-minute treadmill session at 65- 75% of maximal heart rate throughout the eight-week intervention. The HIIT group underwent 15 bouts of 30 seconds at 90-95% maximal heart rate followed by 60 seconds of recovery at an equivalent speed to achieve 50- 55 % of maximal oxygen consumption on a treadmill. In addition, they were encouraged to continue with their regular daily routines but were discouraged to engage in any other form of exercise. They were provided an Omron HJ- 112™ (Bannockburn, IL) pedometer to monitor their daily ambulatory activity.

We used the percentage of VO2max for recovery to determine an accurate speed during the recovery period for each participant considering that 60 seconds is not enough time for the heart rate to decrease and provide an accurate measurement of recovery. We considered using this method to provide a more accurate estimate of recovery intensity.

A qualified trainer was present throughout each session to ensure participants reached and maintained the desired intensity. The speed of the treadmill was adjusted manually while the elevation was maintained constant at 10%. In addition, following their respective interventions, all participants preformed a resistance-training program three times per week following established guidelines ^22^ with a qualified trainer. The purpose of this non- differential co-intervention was simply to introduce participants to the benefits of resistance training. Considering its intensity, we believe this intervention did not have any influence on any of the primary or secondary outcomes ^24^.

To control selection bias and minimize confounding variables, a randomization sequence was generated through four- and six-size permuted blocks with a 1:1 ratio between the groups ^25^. The concealment was made using numbered, sealed envelopes, and the volunteers were assigned to either the HIIT or the MCIT group according to the order of entry in the study. An investigator without direct contact with any of the study participants completed the blinding procedures. Besides, to control information bias, those responsible for recruitment, evaluation, and analysis of the outcome data were blinded to the group assignment and only completed the testing sessions. The staff responsible for conducting the exercise intervention was trained according to the protocols designed for each program. The initial and final evaluations of the outcomes were made at the same time of day, and the interventions were carried out individually ^24^. Identification codes were used for the participants and all the information was stored in file cabinets and password-secured computers only available to the researchers.

In order to determine appropriate sample size, a mean difference in VO2max of 3.5 ml/kg/min with standard deviations (SD) of 2.6 and 4.6 for the HIIT and MICT groups, respectively ^10^, was considered as a minimal difference to reduce cardiovascular disease risk ^3^^,^^4^. We used a 95% confidence level, an alpha error of 5%, and a beta error of 20% assuming a 1:1 ratio between the groups. Using Epidat software (version 4.0), a sample of 20 individuals per group plus 10% for potential losses was calculated.

Intention-to-treat analyses were conducted for comparisons between groups. Also, sensitivity analyses (per protocol analysis) were completed for subjects who completed ≥70% of the training sessions. Normality, homoscedasticity, and linearity tests were considered as basic assumptions for the use of t tests and analysis of covariance (ANCOVA) ^26^ to control for the baseline value of VO2max and adjust for possible confounding variables. Logarithmic transformations and Box-Cox transformations were performed only for secondary outcomes (systolic blood pressure and diastolic blood pressure). However, it was not possible to comply with the parametric assumptions. As summary measures, means and SD were used.

The Mann-Whitney U test was adopted when the assumptions for parametric analyses could not be obtained; in this case, the values are reported in medians and interquartile ranges (IQR). Two-tailed statistical significance tests with a p<0.05 and a 95% confidence level (95% CI) were used. Multiple imputation techniques were applied to the management of missing data for VO2max, systolic blood pressure, and diastolic blood pressure ^27^. All calculations were performed with the Stata software (version 13).

## Results

**Figure 1:**
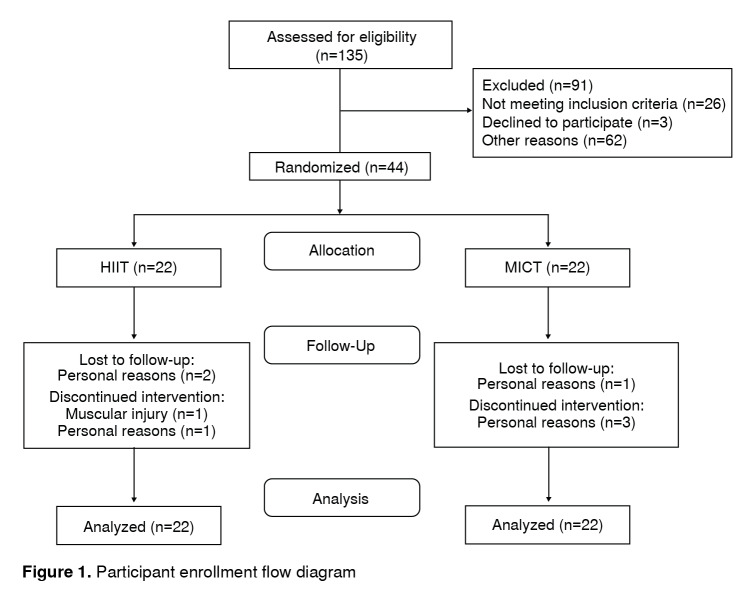
Participant enrollment flow diagram

Data were collected between March, 2015, and May, 2016. A total of 135 individuals who responded to our request to participate were evaluated. Of those who responded, 26 did not meet the selection criteria, three did not agree to participate, and 62 did not enter the study for other reasons (primarily due to time difficulties to comply with the sessions). The final sample consisted of 44 men distributed evenly between the HIIT and MICT groups (N=22 for HIIT group, and N=22 for MICT group) (figure 1).

Participant baseline characteristics are presented in table 1. At baseline, the HIIT group was significantly older and heavier than the MICT group. Besides, the HIIT group had a significantly higher body mass index (BMI), waist circumference, and fat mass (%) than the MICT group. Fat-free mass was significantly higher in the MICT group compared to the HIIT group. In the other variables evaluated, no differences were found between the groups (table 1). Regarding the losses to follow-up, two were reported in the HIIT group and one in the MICT group (figure 1 ). For these three subjects, multiple imputation techniques described above were used and conducted with intention-to-treat analyses.

**Table 1:** Baseline characteristics of study subjects

	HIIT (n=22)	MICT (n=22)	p
Age (years)**	29.5 (25-38)	23.5 (20-34)	0.03‡
Height (cm)*	173.5 (5.79)	171.9 (5.69)	0.36
Weight (kg)**	79.1 (74.6-85.9)	69.3 (63.2-77.4)	0.008‡
BMI (kg/m2)**	26.2 (24.6-27.3)	23.5 (22.0-26.8)	0.03‡
WC (cm)**	87.9 (83.0-91.7)	79.2 (74.7-87.0)	0.006‡
FM (%)*	26.2 (5.6)	20.7 (7.4)	0.008‡
FFM (%)*	35.9 (3.5)	39.7 (5.0)	0.006‡
PAL (Mets/min/week)**	880.0 (540.0-1440.0)	960.0 (360.0-1280.0)	0.91
VO_2máx_ (mL/kg/minutes)*	39.2 (6.0)	42.2 (9.1)	0.20
SBP (mm Hg)**	120.7 (116.0-133.5)	118.2 (116.0-126.0)	0.40
DBP (mm Hg)**	79.2 (76.0-85.0)	77.7 (70.5-87.5)	0.72

BMI: Body mass index; WC: Waist circumference; FM: Fat mass; FFM: Fat-free mass; PAL: Physical activity level; VO2máx: Maximum oxygen consumption; SBP:

Systolic blood pressure; DBP: Diastolic blood pressure

* Values are given as mean ± SD.

** Values are given as medians and interquartile ranges.

‡ Differences between groups at base line, p<0.05

After adjusting for baseline values, age, BMI, weight, and height there was no significant difference in the primary outcome (VO_2_max) between the HIIT and MICT groups (difference (Δ) 0.98 ml/kg/min; 95% CI -2.26 to 4.23, p=0.54) (table 2). VO_2_sub>max increased significantly in those who received the HIIT intervention (39.2 ± 6.0 ml/kg/min vs. 42.7 ± 6.0 ml/kg/min; Δ 3.5 ml/kg/min; 95% CI 2.02 to 4.93; p=0.0001) while in the MICT group, the increase of this variable did not reach significance (VO_2_max change 1.9 ml/kg/min; 95% CI -0.98 to 4.82; p=0.18). When analyzing the VO2max changes individually, it was possible to identify that the participants of the HIIT group presented an average gain of 9.4% vs. 6.0% found in the subjects in the MICT protocol (p=0.67) with a positive intervention response of 81.8% in HIIT compared to 59% for MICT (p=0.09).

In the per protocol analyses (those who completed ≥70% of the programmed training sessions), no differences were found in the primary outcome (VO2max) between groups post-training (HIIT: 44.0 ± 5.8 ml/kg/min vs. MICT: 45.1 ± 8.9 ml/kg/min; p= 0.74). In the intragroup analyses, statistically significant differences and important practical differences were found in both those who received the HIIT intervention (40.4 ± 6.0 ml/kg/min vs. 44.0 ± 5.8; p= 0.0004), as well as in the MICT group (41.7 ± 9.8 ml/kg/min vs. 45.1 ± 8.9; p= 0.03).

Systolic blood pressure and diastolic blood pressure values did not meet the assumption needed to utilize the ANCOVA test, therefore, the non- parametric Mann-Whitney U test was used. In the intention to treat analyses, a lower value of systolic blood pressure was found post-intervention in those in the MICT group (HIIT: 124.5 mm Hg, IQR 120.0-129.5 vs. MICT: 116.5 mmHg, IQR 115.0-119.0); median difference 8 mm Hg (p<0.001). In addition, no significant differences were found after eight-weeks in diastolic blood pressure between the groups (HIIT: 79.2 mmHg, IQR 76.0-85.0 vs. MICT: 79.0 mm Hg, IQR 71.5-83.0); median difference 0.2 mm Hg (p= 0.15) (table 2).

**Table 2:** Effects of HIIT versus MICT on VO2max and systolic blood pressure/diastolic blood pressure after eight weeks: A) Intention-to-treat analysis, and B) per-protocol análisis

A. Intention to treat analysis				
Variables	HIIT (n=22)	MICT (n=22)	Differences between groups (post-intervention)	p
VO_2_máx (ml/kg/min)*	42.7 (6.0)	44.1 (8.7)	0.98 (-2.26 to 4.23)	0.54
SBP (mm Hg)**	124.5 (120.0-129.5)	116.5 (115.0-119.0)	8.0	<0.001‡
DBP (mm Hg)**	79.2 (76.0-85.0)	79.0 (71.5-83.0)	0.2	0.15
**B. Subjects who completed the protocol**				
**Variables**	**HIIT (n=18)**	**MICT (n=18)**	**Differences between groups (post-intervention)**	**p**
VO_2_máx (ml/kg/min)*	44.0 (5.8)	45.1 (8.9)	0.50 (-2.64 to 3.63)	0.75
SBP (mm Hg)**	125.7 (120.0-129.5)	117.2 (115.5-121.0)	8.5	0.0005‡
DBP (mm Hg)**	79.2 (75.0-85.0)	79.2 (71.5-83.0)	0.0	0.26

For maximum oxygen consumption (VO2máx), values were adjusted for base line and confounding variables (age, body mass index, weight and height).

SBP: Systolic blood pressure; DBP: Diastolic blood pressure

* Values are given as mean ± SD.

** Values are given as medians and interquartile ranges.

‡ Differences between groups post-intervention, p<0.05 ANCOVA (95 % confidence interval) for VO2máx Mann-Whitney U Test for SBP and DBP

In the intra-group analyses, no median differences were observed in systolic blood pressure for those who received the HIIT intervention (120.7 mm Hg, IQR 116.0-133.5 vs. 124.5 mm Hg, IQR 120.0 - 129.5; median difference 3.8 mm Hg; p=0.15), or in diastolic blood pressure (79.2 mm Hg, IQR 76.0-85.0 vs. 79.2 mm Hg, IQR 76.0-85.0; p=0.40). In the MICT group, systolic blood pressure was significantly reduced after the intervention (118.2 mm Hg, IQR 116.0-126.0 vs. 116.5 mm Hg, IQR 115.0-119.0); median difference -1.7 mm Hg (p=0.02), while diastolic blood pressure did not change (77.7 mm Hg, IQR 70.5-87.5 vs. 79.0 mm Hg, IQR 71.5-83.0); median difference 1.3 mm Hg (p=0.77).

In the per protocol analyses, the post-intervention values of systolic blood pressure were lower in the MICT group compared to the HIIT group (MICT: 117.2 mm Hg, IQR 115.5-121.0 vs. HIIT: 125.7 mm Hg, IQR 120.0-129.5; p<0.001), whereas for the diastolic blood pressure, the differences did not achieve statistical or practical difference (MICT: 79.2 mm Hg, IQR 71.5-83.0 vs. HIIT: 79.2 mm Hg, IQR 75.0-85.0; p=0.26). In the intragroup analysis of the per protocol analyses, no statistically significant differences were found in the systolic blood pressure and diastolic blood pressure values (table 2).

Training intensities were individually monitored and controlled by trained personnel who continuously supervised the heart rate monitor of each participant during the exercise sessions to guarantee compliance with the intensities for each load. The mean heart rate was determined to be 169.6 beats per minute, equivalent to 91.2% maximum heart rate.

In total, five adverse events (three in the MICT group and two in the HIIT group) occurred during the interventions period, all of which affected the musculoskeletal system. The three events in the MICT group included one event of Hoffitis in the right knee not associated with training in this study, as it was the result of lifting a heavy object at home. The second event was muscle fatigue in the gastrocnemius associated with running on the treadmill, which improved by reducing the training load. The third event was a right medial ankle contusion not associated with the interventions, which occurred while the participant played soccer a day after finishing the training sessions and forced us to postpone the final evaluations for 12 days.

In the HIIT group, there were two adverse events associated with the intervention. The first was right pes anserine bursitis due to training and associated with the inclination of the treadmill. The participant abandoned the intervention after 15 sessions because the symptoms did not improve, despite treatment with oral non-steroidal anti-inflammatories. The second event was tendinitis in the vastus medialis oblique in its insertion in the patella, which was associated with training on the treadmill. The participant partially improved after reducing the training load and medication with non-steroidal anti-inflammatory drugs.

Regarding adherence, 18 individuals in the HIIT group and 19 in the MICT group (81.8% vs. 86.4%, respectively; p=0.71) completed ≥70% of the planned sessions. It should be noted that 13 subjects in the HIIT group and 10 volunteers in the MICT group completed ≥ 90% of the sessions.

## Discussion

Our main finding suggests that after adjusting for confounding variables, such as age, BMI, weight, height, and baseline VO2max, the HIIT protocol was not superior to the MICT protocol in improving the VO2max of this group of apparently healthy young men who engaged in physical activity for less tan 150 minutes/week. This finding is congruent with recent meta-analysis results from apparently healthy young adults reporting that HIIT-based interventions (regardless of their characteristics) did not improve significantly the performance with cardiorespiratory fitness compared to MICT protocols ^17^^,^^28^^,^^29^.

Conversely, from the practical and statistical points of view, those with health impairments, such as classic cardiovascular risk factors (i.e., obesity, hypertension, and blood glucose disorders, among others), coronary heart disease, and heart failure, the HIIT protocols have shown to be more advantageous than the MICT ones to increase VO2max, ^15^^,^^30^^-^^34^. It is noteworthy that in these individuals, an increase of 1.0 ml/kg/min in VO2max has been associated with a reduction in overall mortality, which is considered a clinically significant and relevant change. However, in apparently healthy individuals, an increase of at least 3.5 ml/kg/min in VO max (1 *metabolic equivalent of task*, MET) is required for long-term reductions in mortality and morbidity ^4^^,^^35^^,^^36^.

As it is well known, randomization aims at balancing and accounting for known and unknown factors that could affect the dependent variables; however, it cannot fully guarantee the identification of these factors, especially when sample sizes are small. Therefore, when there are differences between groups in some of the variables, as in this case, they should be adjusted in the final analyses ^37^, which justifies the use of an ANCOVA in our VO2max analyses.

It should be noted that the HIIT group had a baseline VO2max that was 3.0 ml/kg/min lower than the MICT group, which could explain the higher gain obtained by this group (+3.5 ml/kg/min) compared to the increase in the MICT group (+1.9 ml/kg/min). This finding is congruent with previous results revealing that HIIT has an apparent adaptive effect on VO2max in favor of less trained subjects ^30^. Besides, we should mention that individuals in the HIIT group were about six years older than those in the MICT group; yet, despite these differences, participants in the HIIT group were still able to improve VO2max values, although it is well established that VO2max decreases about 10% per decade, regardless of physical activity ^38^. These findings support the notion that HIIT is beneficial to improve aerobic capacity regardless of age and initial fitness levels.

In the intragroup analyses, the VO2max increase was clinically significant in the participants undergoing HIIT, as it changed from 39.2 ml/kg/min at baseline to 42.7 ml/kg/min at the end of the intervention. This finding is similar to that in other studies, which show that a HIIT program improves aerobic power in young, sedentary adults after two and eight weeks of training ^39^ compared to those who do not exercise. Moreover, the intragroup gains in VO2max observed in HIIT averaged 9.4% reaching up to 28.9% while the MICT group only averaged a VO2max increase of 5.9% with increases up to 43.1%.

The improvement in VO2max among those who completed HIIT could be explained by central adaptations including increases in systolic volume and cardiac output, as well as peripheral changes ^40^. Also, peripheral changes, increased number and size of mitochondria, increased mitochondrial enzyme activity, arterial vasodilation, increased nitric oxide bioavailability, and reduced oxidative stress may achieve significant improvements in CRF. It should be noted that although no differences were found in VO2max changes when comparing HIIT vs. MICT, interval training achieved a clinically significant increase in this variable. This benefit occurred with a 7.5-minute stimulus representing 19% of the continuous protocol stimulus and 56% of the total effective exercise time. These findings suggest that important beneficial physiological adaptations could be generated in a shorter amount of time. Besides, in previous studies ^9^^,^^10^^,^^14^^,^^18^^,^^19^, researchers used HIIT interventions with longer load periods and recovery times and fewer or equal intervals compared to this randomized clinical trial.

In regards to the secondary outcome, a lower systolic blood pressure was found in individuals who received the MICT intervention, a difference that achieved statistical and clinical significance. It is worth noting that systolic blood pressure in the HIIT group increased by 3.8 mm Hg compared to its initial value, whereas the MICT group systolic blood pressure was reduced by 1.7 mm Hg from baseline. These changes explain the difference in blood pressures between the groups. Our findings disagree with previous data about the benefits attributed to aerobic exercise, as both HIIT and MICT contributed to the reduction of systolic blood pressure and diastolic blood pressure with higher decreases in hypertensive individuals ^21^.

Nonetheless, no physiological explanations can be provided by the authors to support the increase in systolic blood pressure in the HIIT group other than coincidence, as all the evaluations were performed at the same time of day, under equal conditions, and following the same protocols. This finding differs from that reported in two meta-analyses involving people with cardiovascular risk factors in which no differences in systolic blood pressure were found in those trained with HIIT vs. MICT ^15^^,^^40^.

On the other hand, there is evidence indicating that to improve vascular function, the long-duration HIIT is more effective than the short-duration training ^40^. This could explain the reduction in systolic blood pressure in those receiving the MICT, as the HIIT stimulus only lasted a little over seven minutes.

For diastolic blood pressure, no differences were found between the groups after the intervention or when the baseline and post-intervention values were compared within each of the groups. These results are partially consistent with those found in the meta-analysis by Ramos, *et al.*^40^, who found that postmenopausal women with cardiovascular risk factors achieved a reduction in this blood-pressure component. These results also coincide with the findings in Hwang, *et al.*’s ^15^ meta-analysis reporting no differences in the systolic blood pressure and diastolic blood pressure values in individuals with cardio-metabolic alterations.

While we applied the stimulus with the strength protocol in both groups as a non-differential co-intervention, it is possible that it had some influence on the effect of VO2max, as well as systolic blood pressure and diastolic blood pressure. However, according to the results presented by Buckley, *et al.*^41^ in their randomized clinical trial, when they compared a HIIT protocol against a protocol including HIIT and strength exercises in recreationally active women, they found no statistically significant differences between the groups for VO2max (38.3 ± 4.6 vs. 38.5 ± 5.4 ml/kg/min; p=0.99). Similarly, a recent study among patients with cardiovascular disease compared the effects of a six-month HIIT program and those of an MICT including resistance training during the previous three months and reported no significant improvements in VO2peak for either of the two groups with three and six months of training (HIIT: 28 ± 17 % vs. MICT: 26 ± 29 %; p = 0.824) or systolic blood pressure and diastolic blood pressure (p≥0.05) ^42^. Therefore, we believe our ‘secondary intervention’ did not play a significant role in altering VO2max for any of the groups.

The practical justification for having the strength component in both intervention protocols lies in the need to include strength exercises as a fundamental part of any holistically oriented physical activity program given the importance of incorporating physical fitness, cardiorespiratory endurance, muscular strength, and endurance in exercise intervention programs. Thus, our study focused on improving not only cardiovascular health, but also musculoskeletal health in apparently healthy adults ^22^. Our results may have a greater practical applicability, given that recommendations for physical activity aim to develop the different components of physical fitness, especially the cardiorespiratory and musculoskeletal ones.

A familiarization period before the intervention protocol may be beneficial in reducing the adverse effects of exercise training. Although our study only reported a total of five incidents in the groups, it would be beneficial to reduce such events. The familiarization period could help individuals adjust to the demand of the different loads, given the subjects’ low level of physical activity.

Besides the methodological design used (randomized clinical trial), the main strength of our study was conducting the training sessions in an individualized manner and under the constant supervision of a qualified trainer. Nevertheless, our study is not without limitations: Firstly, we could not control the participants’ physical activity levels beyond the research interventions, which may have affected the variations in VO2max from baseline values and hindered the detection of possible differences between the two groups. Although the volunteers were given a pedometer to monitor their physical activity, it was not possible to record the steps in all of the participants. Another limitation was not using the VO2max baseline values as an inclusion criterion for the volunteers. Although the level of physical activity was used as an inclusion criterion through the Global Physical Activity Questionnaire (GPAQ), some of the individuals who were randomly assigned to the MICT group had VO2max baseline values above the average among subjects with low physical activity levels according to age. Given the characteristics of the interventions in both arms of our study, based on exercise sessions with a group of volunteers exercising at moderate intensity and another group at high intensity, it was difficult to blind participants to the interventions. Finally, we should note that the sample size was calculated with an 80% power.

In summary, the results of this randomized clinical trial do not allow us to affirm that HIIT is superior to MICT for increasing VO2max in healthy 18 to 44-year-old men or vice versa. However, it may be said, as in Milanovic, *et al.*’s ^17^ meta-analysis, that both methods increase VO2max, although when compared none of them shows a more beneficial outcome. As suggested by Gist, *et al.*^28^ and Weston, *et al.*^30^, additional studies are required not only to test the effectiveness of HIIT on VO2max, but also its viability vis-à-vis musculoskeletal limitations, exercise tolerance, and adherence to the protocol. Finally, since most exercise intervention studies have been developed and completed in controlled environments and under constant supervision, it would be very practical to carry out further investigations under less controlled conditions in the context of participants’ daily life.
